# Nomenclatural notes and typification of nine names related to *Jasminum* (Oleaceae)

**DOI:** 10.3897/phytokeys.183.69852

**Published:** 2021-10-12

**Authors:** Bui Hong Quang, Ritesh Kumar Choudhary, Joongku Lee

**Affiliations:** 1 Department of Botany, Institute of Ecology and Biological Resources, Vietnam Academy of Science and Technology, 18 Hoang Quoc Viet, Cau Giay, Hanoi, 100000, Vietnam Institute of Ecology and Biological Resources, Vietnam Academy of Science and Technology Hanoi Vietnam; 2 Graduate University of Science and Technology, Vietnam Academy of Science and Technology, 18 Hoang Quoc Viet, Cau Giay, Hanoi, 100000, Vietnam Graduate University of Science and Technology Hanoi Vietnam; 3 Biodiversity & Palaeobiology Group, Agharkar Research Institute, G.G. Agarkar Road, Pune, Maharashtra, 411004, India Agharkar Research Institute Pune India; 4 Department of Environment and Forest Resources, Chungnam National University, Yuseong-gu, Daejeon 34134, South Korea Chungnam National University Daejeon Republic of Korea

**Keywords:** Jasmine, ICN, Indochina, Nomenclature

## Abstract

Lectotypes are designated here for the following nine validly published names: *Jasminumalongense*, *J.anodontum*, *J.eberhardtii*, *J.harmandianum*, *J.lang*, *J.laxiflorum*, *J.pierreanum*, *J.rufohirtum*, and *J.sinense*. *Jasminumlang* is reinstated as a distinct species.

## Introduction

*Jasminum* L. is a genus of Oleaceae and contains about 200 species distributed widely in tropical and subtropical Asia and the Pacific Islands (Chang et al. 1996; Govaerts and Green 2021). There are 11 species and 2 subspecies of *Jasminum* in Cambodia (Cho et al. 2016), 14 species, 1 variety (1 sp. endemic) in Laos (Green 1999; Newman et al. 2007; Jin et al. 2016), and 40 species, 4 subspecies, and 1 variety (6 spp. endemic) in Vietnam (Ho 2000; Tran 2003; Bui et al. 2016, 2019; Bui 2020).

During our study on the genus *Jasminum* in Indo-China, we could not find holotypes related to nine *Jasminum* names viz. *Jasminumalongense*, *J.anodontum*, *J.eberhardtii*, *J.harmandianum*, *J.laxiflorum*, *J.pierreanum*, *J.rufohirtum*, *J.lang*, and *J.sinense*. Therefore, we have designated lectotypes for these names following Art. 9.3, and 9.11 of the Shenzhen Code (Turland et al. 2018).

## Materials and methods

The following herbaria were consulted either virtually or in-person to trace the type specimens: A, BM, E, HN, IBK, IBSC, K, KUN, LE, MW, NY, P, PE, S, SING, SN, US VNM, and WU (acronyms followed Thiers 2020). Images available on JSTOR Global Plants (https://plants.jstor.org/), Tropicos, International Plant Names Index (IPNI), and Plants of the World Online (POWO) along with relevant monographs and Floras (Gagnepain 1933a, b; Kobuski 1939, 1959; Chang et al. 1996; Green 1993, 1995, 2000, 2003, 2004) were also scrutinized.

## Typification of the names

Gagnepain in Flore Générale de L’Indochine. (Vol. 3), which was published in March 1933, documented 31 *Jasminum* species from the Indochina region and described 11 of them as new to science (1933a). In the same year, he described 13 new *Jasminum* species in Bulletin de la Societe Botanique de France [Vol. 80 (1)] including those 11 species which were already described by him (Gagnepain 1933a; Gagnepain 1933b). To confirm the publication date of the later publication, we contacted Dr. K.N. Gandhi, Sr. Nomenclature Registrar, Harvard University (*Pers. comm.*), who further communicated with the Harvard Botany librarian Ms. Gretchen Wade. The title page of Bulletin Soc. Bot. France vol. 80(1) was found, revealing “The proof for this issue was made May 23, 1933” (English translation). Therefore, we believe the priority of publication goes with Fl. Indo-Chine (Gagnepain 1933a), published in March 1933, and the *Jasminum* names are treated here accordingly.

### 1. Jasminum alongense

Taxon classificationPlantaeLamialesOleaceae

Gagnep., Fl. Indo-Chine [P.H. Lecomte et al.] 3: 1052 (1933a); Bull. Soc. Bot. France 80: 73 (1933b)

082D76D9-F863-5A0F-A9A7-6EF67FA9C54D

#### Lectotype

(here designated).– Tonkin [Vietnam]. baie d’Along, île aux Biches, île près de Hongay, 8 November 1911, *H. Lecomte et A. Finet 840*, P00087775 (P, image!); Isolectotypes: P00087776 (P, image!); [P00087775, P00087776 (K, image!; without any K Barcode)].

#### Nomenclatural notes.

While describing *Jasminumalongense*, Gagnepain (1933a) referred to the specimens collected by H. Lecomte and A. Finet from Tonkin (presently in North Vietnam) Hongay (Hon Gai/ present-day Ha Long Bay). Several duplicates of the same were submitted at P. However, some of them were sent to K, evident from the label on these collections. We could locate two specimens at P (P00087775 and P00087776), and four at K. All K specimens are originally from P, pasted on new sheets and without any K barcode. Two of them, however, possess the preexisting P barcodes P00087775 and P00087776 but the remaining two do not have any). Specimens P00087775 and P00087776 at P are annotated as holotype and isotype respectively, however, they were never published effectively. A thorough survey of literature and virtual herbaria revealed that earlier workers (Ho 2000; Tran 2003) did not designate a lectotype for the name *J.alongense*. Therefore, following Art. 9.6 of the Shenzhen Code (Turland et al. 2018), P00087775 at P is being designated here as the lectotype of *J.alongense*. This specimen not only best matches with the original description but also possesses the reproductive parts and original drawing of the dissected floral parts.

#### Ecology and phenology.

Grows on the Limestone Mountains of the islands of Ha Long Bay. Flowering in November – December, fruiting in March – April.

#### Distribution.

Vietnam, Endemic to Quang Ninh (Ha Long Bay, Hon Gai) Province.

### 2. Jasminum anodontum

Taxon classificationPlantaeLamialesOleaceae

Gagnep., Fl. Indo-Chine [Lecomte et al.] 3: 1040 (Fig. 118) (1933a); Bull. Soc. Bot. France 80: 73 (1933b)

E6D4D2F4-BD21-5A99-A8BF-7D28F4DDEDC2

#### Lectotype

(here designated):–Vietnam. Cochinchine, Bien Hoa, Bruossailles [refers to scrub vegetation], Dong Nai Province, *C. Thorel 949*, P00644260 (P, image!); isolectotypes: P00644261, P00644262 (P, images!).

**Syntypes**. Annam [Vietnam]. Ca-na, Phanrang, 500 m.a.s.l., [Ninh Thuận Province], 12 March 1923, *E. Poilane 9040*, P04046604 (P, image!); Cochinchine [Vietnam]. Bien Hoa, [Dong Nai Province], *C. Thorel 943*, A00105304, A00105305 (A, images!).

#### Nomenclatural notes.

In the protologue of *Jasminumanodontum*, Gagnepain (1933a) referred to two gatherings without citing any field number. The first referred to the collections made by E. Poilane from Ca-na, Phanrang (presently Ninh Thuận) in Annam (present-day Vietnam). Whereas the second referred to the collections made by C. Thorel at Bien-hoa, (Dong Nai Province) from Cochinchine (presently in South Vietnam). However, Gagnepain (1933b), in his other publication, only cited the specimens collected by Thorel viz. *C. Thorel 943* as the original material. We could find this specimen in duplicate at A (A00105304, A00105305), indicated as isosyntypes by P.S. Green. We found some other of Thorel’s specimens P00644260, P00644261, P00644262 (all bearing the same number – *C. Thorel 949*) at P. One of them (P00644260) has the original drawings of the dissected floral parts by Gagnepain. A critical survey of literature and herbarium specimens revealed that earlier workers (Green 2000, 2003; Ho 2000; Tran 2003) did not designate lectotype for *J.anodontum*. Among all of them, we found P00644260 to represent the species best as per the protologue and, therefore, we designate this specimen here as the lectotype of *J.anodontum* following Arts. 9.3 and 9.11 of the Shenzhen Code (Turland et al. 2018). Thorel’s specimens at A are either incomplete or in fruiting, not representing all characters as per the protologue. Therefore, they are designated here as syntypes.

#### Ecology and phenology.

Forest along the roadside, often near the water source. Flowering in January-February, fruiting in March –April.

#### Distribution.

Thailand, Vietnam, (Dak Lak (Dak Mil, Dak Minh), Ninh Thuan (Thuan Nam), Binh Thuan (Phan Thiet), Dong Nai (Bien Hoa City, Vinh Cuu Dong Nai Nature-Culture Reserve). Provinces.

### 3. Jasminum eberhardtii

Taxon classificationPlantaeLamialesOleaceae

Gagnep., Fl. Indo-Chine [Lecomte et al.]. 3: 1051, 1047 (1933a), (Fig.119); Bull. Soc. Bot. France 80: 73 (1933b)

CB9F83D2-CAAC-52BA-B263-CA8B26ED8514

#### Lectotype

(here designated):– Vietnam. Hoa-Binh Province, Mai-Ha, *P.A. Eberhardt 4323*, P00644271 (P, image!); Isolectotype: P00644273 (P, image!).

#### Nomenclatural notes.

*Jasminumeberhardtii* was described (Gagnepain 1933a,b) based on the collections made by P.A. Eberhardt (Coll. no. 4323) from Hoa-Binh Province in northern Vietnam. While searching for the original materials of *J.eberhardtii*, we could locate two specimens P00644271 and P00644273, matching the original description (Art. 9.6 of ICN, Turland et al. 2018), but neither of them was designated as a lectotype (Ho 2000; Tran 2003). Therefore, we selected P00644271, as a lectotype following Arts. 9.3 and 9.11 of ICN (Turland et al. 2018) as it best represents the species. The other specimen (P00644273) is designated as isolectotype.

#### Ecology and phenology.

Grows in the forest between 300–1200 m.a.s.l. Flowering in April – May, fruiting in June – July.

#### Distribution.

Vietnam. Endemic to Hoa Binh (Mai Chau, Mai Ha), and Lao Cai Provinces.

### 4. Jasminum harmandianum

Taxon classificationPlantaeLamialesOleaceae

Gagnep., Fl. Indo-Chine [Lecomte et al.] 3: 1045, 1047 (Fig. 119) (1933a); Bull. Soc. Bot. France 80: 74 (1933b)

CAEEDF3D-E9D8-5533-BF11-F14D3A0F6CD1

#### Lectotype

(here designated):– Laos. *M. Massie s.n.* P00644289. (P, image!); Isolectotype: A00063064 (A, image!).

#### Syntypes.

Cambodia. Expédition du Mékong, Bassac, 1866, *C. Thorel s.n.*, P00644288 (P, image!); K000901473 (K, image!). Vietnam. delta du Mékong: Núi Cam, 01 June 1876, *F.J. Harmand 633*, A00063063 (A, image!); P00644290, P00644291, P00644292 (P, images!).

#### Nomenclatural notes.

*Jasminumharmandianum* was described (Gagnepain 1933a,b) on the basis of three gatherings collected (*Massie s.n.*, *C. Thorel s.n.*, *F.J. Harmand 633*) from Laos, Cambodia (Expédition du Mékong, Bassac) and Vietnam. We found all these elements deposited in P and A, some of them with two duplicates. A survey of literature and virtual herbaria revealed that earlier workers (Green 2000; Ho 2000; Tran 2003) had not designated a lectotype for this species. The specimen P00644289 at P collected by Massie represents species best and has a complete original label. Therefore, it was selected as a lectotype for *J.harmandianum* following Art. 7.11 of ICN (Turland et al. 2018). However, we found a duplicate of the same at A having barcode A00063064 which was distributed from P, as evident from its label. It is designated here as an isolectotype.

#### Ecology and phenology.

Grows in the forest at 100–1000 m.a.s.l. Flowering in June – July, fruiting in August – September.

#### Distribution.

Cambodia, Laos, Thailand and Vietnam. Dak Lak (Dak Mil), Kon Tum (Chu Mon Ray National Park), and An Giang (Nui Cam) Provinces.

### 5. Jasminum lang

Taxon classificationPlantaeLamialesOleaceae

Gagnep., Fl. Indo-Chine [Lecomte et al.]. 3: 1046 (1933a); Bull. Soc. Bot. France 80: 77 (1933b)

FBCA8D4B-5BCA-5053-B84C-F1399D3AC9DE

#### Lectotype

(here designated):– Vietnam. Kiên Khê, in collib. Dong Bau, (Ha Nam Province), 31 March 1885, *H.F. Bon 2869*, P00640614 (P, image!); Isolectotype: K000901460 (K, image!).

#### Syntypes.

Vietnam. Kiên Khê, in collib. Dong Bau, (Ha Nam Province), 19 March 1884, *H.F. Bon 2604*, P00640615 (P, image!); Presqu’ile de Nui-han-heo (Hon Heo mountain, currently in Ninh Hoa Town, Khanh Hoa Province), 12 June 1933, *Poilane 6867* (K, image!), VNM00021039 (VNM, image!).

#### Nomenclatural notes.

*Jasminumlang* was described (Gagnepain 1933a,b) based on three gatherings (*H.F. Bon 2604*, *2869 and Poilane 6867*) collected from north, and south Vietnam. We found two duplicates matching the detailed description and specimen label information. A survey of literature and multiple herbaria revealed that a lectotype for *J.lang* had not been designated in earlier studies (Ho 2000; Tran 2003). We, therefore, selected the P specimen (P0040614) as a lectotype for *Jasmniumlang* following Arts. 9.3 and 9.11 of ICN (Turland et al. 2018) as it represents the species best. The other specimen at K (K000901460) is designated here as isolectotype.

#### Taxonomic notes.

*Jasminumlang* is known from China and Vietnam (Gagnepain 1933a,b; Chia 1952; Miao 1984; Chang et al. 1996; Ho 2000). During the course of our revisionary work, we critically compared the morphological characters of *Jasminumlang* with *Jasminumcoffeinum* Hand.-Mazz. because of their morphological differences and their treatment as synonyms (Chang et al. 1996; Green 2006). Our field observation and study of the multiple specimens housed in various national and international herbaria made us realize that the species are morphologically different. Indeed, the characters used for their delimitation (Handel and Heinrich 1925; Gagnepain 1933a,b; Chia 1952; Miao 1984; Chang et al. 1996; Ho 2000), as indicated in Table 1, clearly indicate that they are different entities. Therefore, we reinstate *J.lang* Gagnep., as a distinct species.

**Table 1.** Morphological differences between *Jasminumlang* and *J.coffeinum.*

Morphological characters	*Jasminumlang*	*Jasminumcoffeinum*
Branchlets	terete or compressed	terete or 4-angled, narrowly winged
Number of secondary veins in leaf	7‒11	5‒9
Petiole length	0.8‒2.5 cm long	1‒2 cm long
Inflorescence	solitary, terminal or axillary 2‒6 flowered	subopposite or fascicled in leaf axils, 3‒10 flowered
Bracts	linear, 5‒11 mm	ovate or spatulate, 2‒5 mm
Calyx	glabrous, tube 2.8‒4 mm; lobes 6‒8, linear, 0.5‒1.8 cm, enlarged to 2‒3 cm in fruit	puberulent, tube ca. 4 mm; lobes 5, narrowly deltate, 1‒2 mm, not enlarged in fruit
Corolla	white, pink outside, lobes narrowly lanceolate, 2‒2.5 cm	white, lobes lanceolate, 1‒1.2 cm

#### Ecology and phenology.

Grows in the forest at altitude 100–800 m.a.s.l. Flowering in February – April, fruiting May – June.

#### Distribution.

Vietnam. Endemic to Hoa Binh (Mai Chau), Ha Nam (Kien Khe), Quang Ninh (Ha Long bay), and Khanh Hoa (Hon Heo, Hon Ba Nature Reserve) Provinces.

### 6. Jasminum laxiflorum

Taxon classificationPlantaeLamialesOleaceae

Gagnep., Fl. Indo-Chine [Lecomte et al.] 3: 1055 (1933a); Bull. Soc. Bot. France 80: 75 (1933b)

8F222543-31E2-51C1-9BE2-A5A59BA82048

#### Lectotype

(here designated):– Vietnam. Austro-Cochinchine, Tanh-huyen, Thu-duc, Baria, Bien-hoa (Dong Nai Province), 01 December 1868, *L. Pierre 327*, P03868745 (P, image!); Isolectotypes: A00105308, A00105309, A00105310, A00105313 (A, images!); BM000997658 (BM, image!); P03868746, P03868747, P03868750 (P, images!).

#### Syntypes.

Vietnam. *C. Thorel 862*, A00105593 (A, image!); P03868756, P03868755 (P, images!); S09–37061 (S, image!); Phu-mi, 1875 *Godefroy s.n.* P03868749 (P, image!); 26 January 1903, Phu-mi, *D.G.J.M. Bois 2180*, A00105307 (A, image!); Phu-mi, P03868743 (P, image!); Bien-hoa à Xuan-loc (Dong Nai Province), 12 January 1914, *A. Chevalier 29979*, P03868744 (P, image!); Nui dinh pro de Baria (Ba Ria-Vung Tau Province), 100 m, 27 October 1919, *E. Poilane 671*, A00105311 (A, image!), P03868753, (P, image!); environs de Saigon (Ho Chi Minh city), 28 January 1926, *F. Evrard 2618*, *coll. Khai 161*, P03868742 (P, image!); km. 80.500 de la route col. N°20 pro: du Haut Donaï délégation de Djyrinh (Dinh Linh, Lam Dong Province), 19 October 1931, *E. Poilane 19787*, P03868752 (P, image!); A00105312, (A, image!).

#### Nomenclatural notes.

*Jasminumlaxiflorum* was described (Gagnepain 1933a,b) based on seven gatherings (*E. Poilane 19787*, *F. Evrard 2618*, *coll. Khai 161*, *C. Thorel 862*, *L. Pierre 327*, *A. Chevalier 29979*, and *Godefroy s.n*) collected from South Vietnam. None of them was designated as a holotype. We found nine specimens matching the original description and information given on the labels which are included in the protologue, and several duplicates. A survey of the literature (Ho 2000; Tran 2003) and multiple herbaria revealed that a lectotype for *J.laxiflorum* has not yet been designated. Therefore, *L. Pierre 327*, the specimen P03868745 at P, which best represents the species, is designated here as a lectotype following Arts. 9.3 and 9.11 of ICN (Turland et al. 2018). Other specimens at A, P, and BM (A: A00105308, A00105309, A00105310, A00105313; BM: BM000997658; P: P03868746, P03868747, P03868750) are designated here as isolectotypes.

#### Ecology and phenology.

Grows in the forest at 100–500 m.a.s.l. Flowering in February –March, fruiting April – May.

#### Distribution.

Vietnam. Endemic to Dak Lak (Dak Mil), Kon Tum (Chu Mon Ray National Park), Lam Dong (Di Linh), Dong Nai (Bien Hoa, Trang Bom) Ba Ria-Vung Tau (Ba Ria nui dinh), and Ho Chi Minh City (Thu Duc) Provinces.

### 7. Jasminum pierreanum

Taxon classificationPlantaeLamialesOleaceae

Gagnep., Fl. Indo-Chine [Lecomte et al.] 3: 1042 (1933a); Bull. Soc. Bot. France 80: 76 (1933b)

70712043-A4BB-5629-99D7-F2A703362899

≡ Jasminum rarum Kerr, Bull. Misc. Inform. Kew 28 (1938). Type: Thailand. Southwestern, Kanchanaburi, Bau Ke, Thailand, Kanchanburi Bau Ke, 21 July 1926, Put, *N.211* (holotype: K000901476 (K, image!); isotypes: E00284807 (E, image!, L0005365 (L, image!, P00644299 (P, image!).
≡ Jasminum cordatulum (Merr. & Chun ex L.C.Chia) L.C.Chia, Fl. Hainan. 3: 576 (1974). Type: China. Hainan Province, 8 September 1933, *Liang Xiangri 62960* (holotype: IBK IBK00191326 (IBK, image!; isotypes: IBK00191326 (IBK, image!), PE00027990 (PE, image!).
≡ Jasminum seguinii var. cordatulum Merr. & Chun ex L.C.Chia, Acta Phytotax. Sin. 2: 43 (1952). Type: China. Hainam, 26 August 1933, *Liang Xiangri 62823* (holotype: IBK00093762 (IBK, image!); isotypes: IBSC0459241(IBSC, image!), PE01489419 (PE, image!, SN005501 (SN, image!).

#### Lectotype

(here designated):– Vietnam (Cochinchine). Prope Chiao Xhan ar originum fluvii Dongnai septentrione Prov. Bien hoa “Bois Hua”, March 1877, *L. Pierre 2828*, P00640601 (P, image!); Isolectotypes: P00640600 (P, image!), VNM00021049 (VNM!).

#### Syntype.

Cambodia. monts Cherreer, June 1870, *L. Pierre s.n.*, P00644300 (P, image!).

#### Nomenclatural notes.

*Jasminumpierreanum* was described (Gagnepain 1933a,b) based on two gatherings (*L. Pierre 2828* and *L. Pierre s.n*) collected from Cambodia and Vietnam. We found three duplicates matching the detailed description and specimen label information included in the protologue. A survey of literature and virtual herbaria revealed that a lectotype for *J.laxiflorum* has not been designated in earlier studies (Green 2000, 2003; Ho 2000; Tran 2003). We, therefore, selected the P specimen (P00640601) as a lectotype which best matched the description, following Arts. 9.3 and 9.11 of ICN (Turland et al. 2018). Other specimens at P and VNM (P00640600 and VNM00021049) are designated as isolectotypes.

#### Ecology and phenology.

Grows in the forest at high altitude 300–1000 m.a.s.l. Flowering in August – September, fruiting in January – February.

#### Distribution.

Cambodia, China, Thailand and Vietnam. Hoa Bình (Mai Chau), Thua Thien-Hue (Phu Loc), Gia Lai (Kbang), Dak Lak (Dak Mil), and Dong Nai (Bien Hoa) Provinces.

### 8. Jasminum rufohirtum

Taxon classificationPlantaeLamialesOleaceae

Gagnep., Fl. Indo-Chine [Lecomte et al.]. 3: 1057 (1933a); Bull. Soc. Bot. France 80: 77 (1933b)

041CC524-FA38-5EA3-85CD-5D3BFB05967B

≡ Jasminum yunnanense Z.P.Jien ex P.Y.Pai, Acta Bot. Yunnan. 5: 66 (1983). Type: China. Yunnan, 18 April 1956, *Sino-Soviet Yunnan Joint Investigation Group* 955 (holotype: KUN0548902 (KUN, image!; isotypes: PE00027944, PE01501575, PE01501578 (PE, images!, IBSC0460998 (IBSC, image!).

#### Lectotype

(here designated):– Vietnam (Tonkin). Province de Sonla, Canton de Muong Mua Chan De Mai Son, 540 m, 26 May 1927, *Pételot 5032*, P00644294 (P, image!); Isolectotypes: P00644295 (P, image!), NY00297220 (NY, image!).

#### Syntype.

Laos. s.loc. *H. d’ Orléans s.n.*, P00644296 (P, image!).

#### Nomenclatural notes.

*Jasminumrufohirtum* was described (Gagnepain (1933a,b) based on two gatherings (*Pételot 5032* and *H. d’ Orléans s.n*) collected by Pételot and H. d’ Orléans from north Vietnam and Laos respectively. While tracing the holotype, we found two duplicates matching the detailed description and specimen label information which are included in the protologue. A survey of literature and virtual herbaria revealed that a lectotype for *J.rufohirtum* has not been designated by the earlier workers (Miao 1992; Chang et al. 1996; Ho 2000; Tran 2003; Prachaya et al. 2004). We selected the P specimen P00644294 as a lectotype following Arts. 9.3 and 9.11 of ICN (Turland et al. 2018) as it represents the species best. The other specimens at P, and NY, are designated here as isolectotypes.

#### Ecology and phenology.

Grows in the forest at altitude 200–1000 m.a.s.l. Flowering in April – May, fruiting May – July.

#### Distribution.

China, Laos, Thailand and Vietnam. Son La (Muong Ma), Dien Bien (Muong Cha, Ta Chua), and Lao Cai (Bat Xat Nature Reserve) Provinces.

### 9. Jasminum sinense

Taxon classificationPlantaeLamialesOleaceae

Hemsl., J. Linn. Soc., Bot. 26: 80 (1889)

77320E2A-4B4B-5D87-8110-3D2769D3BF1C

≡ Jasminum bodinieri H.Lév.Repert. Spec. Nov. Regni Veg. 13: 151 (1914). Type: China. Environs de Tsin-Gay, Gan-Pin./Item envir. de Tou-Chan. Juillet 97 J. Cavalerie, 15 July 1897, *E.M.Bodinier & J.P.Laborde 1890*, Type: (syntype: E00284832 (E, image!). Environs de Kouy-yang. Mont du College, 11 September 1898, *Bodinier E.M.*, *1890* (syntype: E00284833 (E, image!).
≡ Jasminum sinense var. septentrionale Hand.-Mazz., Symb. Sin. 7: 1012 (1936). Type: China. Yunnan: Prov. Yünnan bor.-occid.: In silva frondosa subtropica inter vicos Tjiontson et Pipiti ad fluvium Lu-djiang (Salween) infra Tschamutong. 1700 m. asl., 17 August 1916, *Handel-Mazzetti*,*H.R.E. von*, *Handel-Mazzetti*, Iter sinense 9848 (holotype: WU0060941 (WU, image!).
≡ Lonicera cavaleriei H. Lév. Repert. Spec. Nov. Regni Veg. 11(271–273): 31 (1912). Type: China. Pan-choui, route de Pin-Fa Tou-Yun., 9 April 1907, *Cavalerie J.*, *3038* (holotype: E00284831 (E, image!).

#### Lectotype

(here designated):– China. Hupeh [Hubei] Nan-T’o and mountains to Northward, 1887, *A. Henry 4464*, K000901325 (K, image!); Isolectotype: K000901323 (K, image!).

#### Syntypes.

China. Hupeh [Hubei] Nan-T’o and mountains to Northward, 1887, *A. Henry 2106*, K000901324 (K, image!), US00112856 (US, image!); Kwantung [Guangdong], North river, August 1887, *Ford 114*, K000901326 (K, image!), P00640606, P00640607 (P, images!), IBSC0002797 (IBSC, image!).

#### Nomenclatural notes.

Hemsley (1889) described *Jasminumsinense* based on three gatherings (*A. Henry 2106*, *4464* and *Ford 114*) from China (Hubei and Kwangtung Province). Green (1993) in one of his publications, cited these gatherings as syntypes. A survey of literature and multiple herbaria revealed that a lectotype for *J.sinense* has not been designated in earlier studies (Green 1993, Chang et al. 1996, and Bui et al. 2013). While looking for the original materials, we found eight specimens of these collections deposited in IBSC, K, P and US (IBSC0002797, K000901323, K000901324, K000901325, K000901326, P00640606, P00640607 and US00112856). One of the sheets at K holds Henry’s two gatherings 4464 and 2106, together with barcodes K000901323 and K000901324, respectively. We, therefore, selected Henry’s collection (No. 4464; K000901325) at K as lectotype because it represents the species best and has a complete original label, following Arts. 9.3 and 9.11 of ICN (Turland et al. 2018). Although K000901323 is also a complete and flowering specimen, it was selected as an isolectotype to avoid any confusion which the two gatherings on the same sheet (K000901323 and K000901324) may create. The remaining specimens are designated here as syntypes.

#### Ecology and phenology.

Grows in the forest at a high altitude of 800–2000 m.a.s.l. Flowering in June – August, fruiting in September – November.

#### Distribution.

China and Vietnam. Ha Giang Province (Pho Bang).

## Supplementary Material

XML Treatment for Jasminum alongense

XML Treatment for Jasminum anodontum

XML Treatment for Jasminum eberhardtii

XML Treatment for Jasminum harmandianum

XML Treatment for Jasminum lang

XML Treatment for Jasminum laxiflorum

XML Treatment for Jasminum pierreanum

XML Treatment for Jasminum rufohirtum

XML Treatment for Jasminum sinense
